# Next Generation Sequencing-Based Germline Panel Testing for Breast and Ovarian Cancers in Pakistan

**DOI:** 10.31557/APJCP.2021.22.3.719

**Published:** 2021-03

**Authors:** Hassan Tariq, Asma Gul, Tahir Khadim, Hafeez Ud-Din, Hamid Nawaz Tipu, Muhammad Asif, Rabia Ahmed

**Affiliations:** 1 *Department of Histopathology, Armed Forces Institute of Pathology, Rawalpindi, Pakistan. *; 2 *Department of Immunology, Armed Forces Institute of Pathology, Rawalpindi, Pakistan. *; 3 *Department of Histopathology, PNS Shifa Hospital, Karachi, Pakistan. *

**Keywords:** BRCA1/2, triple negative breast cancer, high grade epithelial ovarian cancer, second generation sequencing

## Abstract

**Background:**

Pathogenic germline mutations in *BRCA1/2* constitute the majority of hereditary breast and/or ovarian cancers worldwide. Incidence and mortality rate of breast and ovarian cancers in Pakistani women is high. Thus, to establish the diagnosis for targeted therapy in Pakistan, we conducted Next-generation sequencing-based germline testing for the detection of *BRCA1/2* oncogenic variants associated with breast and ovarian cancer subtype.

**Methods:**

Peripheral blood of 24 women, diagnosed with breast and epithelial ovarian cancers, was taken from the recruited cases with the consent of performing germline genetic testing. DNA was isolated from the peripheral blood and subjected to indexed BRCA Panel libraries. Targeted NGS was performed for all coding regions and splicing sites of *BRCA1* and *BRCA2* genes using AmpliSeq for Illumina BRCA Panel and Illumina MiSeq sequencer (placed at AFIP). Analysis of the sequencing results has been done by using Illumina bioinformatics tools.

**Results:**

We detected 421 variants having a quality score of 100 in all cases under study. The list of identified variants in* BRCA1* and *BRCA2* genes was narrowed down after filtering out those which did not pass q30 and those with a minor allele frequency (MAF) > 0.05 based on gnomAD browser. To classify these variants, clinical significance was predicted using external curated databases. As a result, we interpreted (n = 4) 16.7% pathogenic variants in *BRCA1* and (n = 6) 25% variants of uncertain significance (VUS) in both genes. Descriptive statistics depicted that the age and BMI of BRCA positive cases are less than BRCA negative cases.

**Conclusion:**

Our findings exhibit an initial report for the NGS based cancer genetic testing in Pakistan. This will enable us to pursue screening and diagnosis of hereditary BRCA mutation utilizing the latest state-of-the-art technique locally available in Pakistan ultimately resulting in targeted cancer therapy.

## Introduction

In spite of rigorous research and considerable progress in precise diagnosis for the prediction of targeted therapy of breast and ovarian cancers worldwide, these malignancies remain a major health problem and are a predominant cause of death in women. Studies have shown that 10% to 15% of ovarian cancer cases and 5% to 10% of breast cancer cases are inherited, caused by anomalous genes passed from parent to offspring (Fackenthal and Olopade, 2007: Ramus and Gayther, 2009). *BRCA1* and *BRCA2* are tumor-suppressor genes and associated with DNA damage repair and recombination, control for the cell-cycle checkpoint, transcriptional regulation, and apoptosis (Venkitaraman, 2014). Deleterious mutations in *BRCA1/2* genes, associated with hereditary breast and ovarian cancer syndrome, run in autosomal dominant pattern with variable penetrance (Miki et al., 1994; Wooster et al., 1995; Ripperger et al., 2009). 

Research has provided evidences that in *BRCA1* and *BRCA2* mutation carriers peak breast cancer onset is approximately at 30-40 and 40-50 years of age, respectively. Morbidity rate after cancer onset remains constant at around 25% per year until the age of 80 years (Kuchenbaecker et al., 2017). Consequently, *BRCA1/2* were selected as a biomarker for individual diagnostics and prediction for therapeutics. Previous investigations have confirmed that the *BRCA*-mutated cancer patients are sensitive to platinum chemotherapy. These cases show a good prognosis after treatment with poly (ADPribose) polymerase inhibitors (Vidula and Bardia, 2017; Taylor and Eskander, 2018). Food and Drug Administration (FDA) has approved the poly (ADP-ribose) polymerase inhibitors that target and kill HER2 negative *BRCA* mutated cancer cells (Beniey et al., 2019). *BRCA1* gene is located on 17q21, and contains 24 exons that code for 1863 amino acids (NM_00,724.3), (Miki et al., 1994) whereas *BRCA2* gene is located on 13q12e13, and contains 27 exons that code for 3,418 amino acids (NM_000059.3) (Wooster et al., 1995). In BRCA, there are no evident mutation hot spots except in the Ashkenazi population (Roa et al., 1996; Janavičius, 2010). 

Breast cancer is the most prevalent type in the world of all cancers and in Pakistani women too. It is the second leading cause of death among women. As per WHO factsheet, in Pakistan breast cancer ranked first on incidence, mortality and prevalence by cancer site, and ovarian cancer is ranked tenth on incidence and twelfth on death rate (GLOBOCAN, 2019). And according to National Comprehensive Cancer Network (NCCN) guidelines, breast carcinoma patients with triple negative immunohistochemical markers; Estrogen, Progesterone and HER2/ Neu, with age equal or less than 60 should be screened for *BRCA* mutations/variants. 

Ovarian cancer is leading in gynecologic malignancies in women. There is widespread agreement that all women diagnosed with epithelial ovarian cancers (EOC), with family history of cancer, should be referred for cancer genetic counseling and recommended for germline *BRCA1/2 *mutations testing. (NCCN , 2019). Among different EOC subtypes, the prevalence of BRCA mutation varies. It is reported up to 20-25% in High grade serous carcinoma, the highest frequency among EOC subtypes. (Hennessy et al., 2010; Ledermann et al., 2016). In endometroid carcinoma and other EOC subtypes, *BRCA *mutation was reported<10% (Arts-de Jong et al., 2016). 

Sanger sequencing is often performed for BRCA testing, considered as the gold standard of DNA sequencing. Even so, lower cost-effectiveness and limited throughput are disbenefits of Sanger sequencing which restrict the development of this technique in clinical genetic testing. For now, the advancement of DNA sequencing technologies has equipped unparalleled opportunities for researchers to detect and investigate the significance of genetic variations in common disorders. Next-generation sequencing (NGS) has been integrated in the clinical diagnostics for *BRCA *mutation testing with the edge of high-throughput testing. Clinical studies and research on BRCA testing have depicted that NGS offers cost-effectiveness high specificity and sensitivity in comparison with current approaches (Walsh et al., 2010; Chan et al., 2012, Qu et al., 2019). The American College of Medical Genetics and Genomics (ACMG) guidelines indicate the classification of variants into five categories: pathogenic, likely pathogenic, variant of uncertain significance (VUS), likely benign, and benign. Only clinically significant variants are considered to be pathogenic or likely pathogenic. Reporting of VUS is still not uniform in all laboratories worldwide. Mostly, the diagnostics labs report VUS detected in the gene in clinical question, for example a VUS in *BRCA1* gene detected in a breast cancer patient (Richards et al., 2015; Li et al., 2017; Vears et al., 2017).

To date, no NGS-based genetic testing of breast and ovarian cancer has been performed and reported indigenously rather the sample are outsourced to foreign laboratories. The primary aim in this study is to establish NGS system in a clinical laboratory i.e. Armed force institute of pathology (AFIP) Pakistan, for the genetic testing of breast and ovarian cancer subtypes. In the present study, all exons and splice site regions of *BRCA1* and *BRCA2 *genes were screened by targeted next generation sequencing with the objective of detecting germline pathogenic variants cases under study. 

## Materials and Methods

*Enrollment of families*

Cases were recruited among the patients diagnosed with epithelial ovarian cancers and breast carcinomas at histopathology department of Armed force institute of pathology (AFIP) Rawalpindi, Pakistan from November 2019 to February 2020. Early onset breast cancer and triple negative breast cancer patients aged less than 60 were included. Ovarian cancer patients with high grade serous carcinoma and endometroid carcinoma were included. This project was approved by the Ethical Committee of the Armed forces Institute of Pathology (AFIP), Pakistan. Each study participant was interviewed and asked to share their family history and demographic information and to sign an informed consent for genetic testing. 

*DNA isolation and Sequencing*

Peripheral blood samples along with the consent and family history were taken from the recruited study participants. By using GeneJET Genomic DNA Purification Kit (ThermoFisher, USA), genomic DNA was extracted from whole blood to carry out germline pipeline for mutation testing. Isolated DNA was quantified by using Qubit assay kit (Thermo Fisher Scientific, USA). DNA was diluted according to the input recommendation of AmpliSeq for Illumina BRCA Panel for library preparation. Coding and splice-site regions of *BRCA1 *and *BRCA2 *genes (NM 007294 and NM 000059 respectively) were amplified and uniquely indexed according to the AmpliSeq for Illumina workflow and were proceeded to paired-end sequencing by synthesis with the minimum 500X coverage depth. Target capture and bridge amplification were carried out. Then signal imaging and extension were achieved in the automated 300 cycles on the clusters- bearing V2 flow cell utilizing MiSeq sequencer (Illumina, San Diego, CA, USA). Eventually, the raw reads were analysed to measure the base quality and amplicon coverage. 

*Data analysis*

Local run manager of MiSeq sequencer aligned the cleaned reads to the human reference genome hg19/GRCh37 and subsequently called mismatched reads as variants by using Illumina’s integrated bioinformatics tools. Genetic variants were identified using Illumina’s Basespace sequence hub variant caller, based on Pisces 5.2.9.23. Annotation of variants was performed using Basespace variant interpreter build on Annotation Engine 3.1.1.0. 

*Variant classification*

As per the guidelines of American College of Medical Genetics and Genomics and the Association for Molecular Pathology – ACMG/AMP (Richards et al., 2015), variant interpretation was carried out. To categorize the variants into pathogenic, likely pathogenic, variant of uncertain significance (VUS), likely benign or benign; in-silico prediction software and external curated databases were used. On the basis of the output of programs, SIFT (http://sift.jcvi.org/), PolyPhen-2 (http://genetics.bwh.harvard.edu/pph2/) and MutationTaster (http://www.mutationtaster.org/), functional effect of resultant variants on the protein of *BRCA1* and *BRCA2* genes were identified. For minor allele frequencies of resultant variants, genome aggregation database (https://gnomad.broadinstitute.org/) was used. ClinVar (https://www.ncbi.nlm.nih.gov/clinvar/), dbSNP (https://www.ncbi.nlm.nih.gov/snp/), The BRCA Exchange (https://brcaexchange.org) and Genomic Data Commons Data Portal (https://portal.gdc.cancer.gov/) databases were consulted for the respective variants to examine their clinical significance. For descriptive statistics, SPSS Statistics 27 was used to compare oncogenic, uncertain and benign variants in *BRCA1/2*. 

## Results

*Cases*

**Figure 1:**
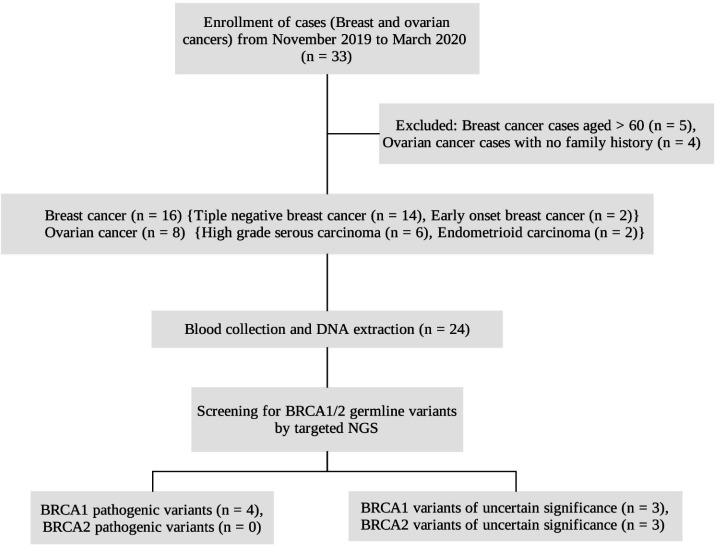
Flow Diagram Depicting the Enrollment of Study Participants, Screening Methodology Used and *BRCA1/2 *Variants Detected

In this study, sample size was dependent on reagents and consumables available by limited resources. Thus, we carried out 24 cases for targeted sequencing on germline panel. Cases diagnosed with Triple negative breast cancer, Early onset breast carcinoma, High grade serous carcinoma and Endometrioid carcinoma were included. Enrollment of cases is shown in Figure 1. Mean Age at diagnosis and Body mass index (BMI) of the recruited study participants were 48.6 and 27.3 respectively. 

*Sequencing and variant interpretation*

*BRCA1 *and *BRCA2* sequencing of 24 cases resulted in 421 variants having variant quality score of 100. Coverage of sequencing reads and average Quality score (Q30) and were 96.6% and 95% respectively. Consequence of variants were missense, nonsense, indels, and synonymous in exonic region and also detected variants in 5’UTR and splice site regions. The list of identified variants was narrowed down after excluding those with a minor allele frequency > 0.05 based on gnomAD browser. Besides in-silico analysis, clinical significance of the resultant variants was consulted with external databases as mentioned in methodology. From variant calling files of all cases, we identified 4 pathogenic variants (0.95%) in *BRCA1* and 6 variants of uncertain significance (1.43%) in *BRCA1* and *BRCA2* with miner allele frequency ≤ 0.05 and 97% variants were benign. Details of these reportable variants along with the family history of respected cases are mentioned in Table 1 and the descriptive statistics are presented in Table 2. 

*BRCA1 variants*

A stop gained mutation in *BRCA1 p.Arg1443Ter, dbSNP ID *is* rs41293455*, population allele frequency is 0.00 in South Asia, identified in the youngest patient aged 31 diagnosed with triple negative breast cancer without any family history of cancer. This variant is terminating translation and causing truncated *BRCA1 *protein. Another pathogenic variant in a TNBC case, a frameshift deletion mutation *p.Ser956ValfsTer13*, *rs80357819*, in *BRCA1 *is detected in a case aged 32 with no family history of cancer. Two cases of high grade serous carcinoma aged 36 and 70, with family history of cancer, have been detected of *BRCA*1 pathogenic variants, frameshift duplication *p.Tyr655ValfsTer18* (*rs80357522*) and missense *p.Arg1203Ter* (*rs62625308*) respectively. Variants of uncertain significance in *BRCA1* are detected in three cases of early onset breast cancer with the family history of male breast cancer (*rs753888336*), triple negative breast cancer with no family history of cancer (*rs775417240*) and a high grade serous carcinoma with family history of breast cancer (*rs753888336*). 

*BRCA2 variants*

In this study, no oncogenic variants are detected in *BRCA2* gene however variants of uncertain significance are identified in two individuals of triple negative breast cancer, one case with family history of cancer and the other one has not (*rs587781399*, *rs431825354*), and a VUS in high grade serous carcinoma with family history of thyroid cancer (*rs80359254*).

**Table 1:** Details of Breast and Ovarian Cancer Cases with Reportable Germline *BRCA* Mutations

Age (years)	Gene	Variant^a^	MAF^b^	Zygosity (VAF^d^)	Variant classification^e^	Cancer	Family history
31	*BRCA1*	Stop gained c.4327C>T p.Arg1443Ter	Global= 0.000025, SA^c^= 0.00	Heterozygous (0.52)	Pathogenic	Triple negative breast cancer	_
32	*BRCA1*	Frameshift deletion c.2866_2870delTCTCA p.Ser956ValfsTer13	_	Heterozygous (0.49)	Pathogenic	Triple negative breast cancer	_
36	*BRCA1*	Frameshift duplication c.1961dupA p.(Tyr655ValfsTer18)	Global= 0.00001197, SA= 0.000098	Heterozygous (0.44)	Pathogenic	High grade serous neoplasm	Breast cancer (Sister)
70	*BRCA1*	Missense c.3607C>T p.Arg1203Ter	Global= 0.00001195, SA= 0.00	Heterozygous (0.45)	Pathogenic	High grade serous neoplasm	Ovarian cancer (Sister)
31	*BRCA1*	Intronic c.4358-2789A>G	_	Heterozygous (0.49)	VUS	Early onset breast carcinoma	Male breast cancer (Uncle)
38	*BRCA1*	Inframe deletion c.5571_5579delGATCCCCCA p.Gln1857_Pro1859del	_	Heterozygous (0.50)	VUS	Triple negative breast cancer	_
54	*BRCA1*	Intronic c.4358-2789A>G	_	Heterozygous (0.49)	VUS	High grade serous neoplasm	Breast cancer (paternal cousin)
33	*BRCA2*	Missense c.7628A>G p.(Tyr2543Cys)	Global= 0.00003189, SA=0.000196	Heterozygous (0.48)	VUS	Triple negative breast cancer	_
45	*BRCA2*	Missense c.1070A>C p.(Glu357Ala)	Global= 0.00004414, SA= 0.000365	Heterozygous (0.582)	VUS	Triple negative breast cancer	Breast, skin, prostate cancers (Paternal cousins) Breast cancer (Mother and 2^nd^ cousin of mother)
71	*BRCA2*	Missense c.9934A>G p.(Ile3312Val)	Global= 0.00001592, SA= 0.00	Heterozygous (0.47)	VUS	High grade serous neoplasm	Thyroid cancer (Sister)

^a^, Variant nomenclature according to HGVS (Human Genome Variation Society) standards; ^b^, Minor allele frequency (MAF) indicates the least frequent allele at a specific locus in a given population. For MAF, genome aggregation database (https://gnomad.broadinstitute.org/) was used. ^c^, SA is abbreviated form of South Asian; ^d^, VAF is variant read frequency, ratio of the observed sequence reads matching a specific DNA variant at a locus to the overall coverage at that locus. It is a measure of variant proportion present in the original sample describing zygosity; ^e^, Interpretation was done by utilizing ACMG guidelines, curated external databases and resources i.e. dbSNP, ClinVar, GDC Data portal, The BRCA Exchange

**Table 2:** Characteristics of Cases under Study with Descriptive Statistics

	Total cases (n = 24)	*BRCA* positive (n = 4)	*BRCA* uncertain (n = 6)	*BRCA* negative (n = 14)
Age: Mean (SD)	48.6 (14)	42.2 (18.6)	45.3 (15.1)	51.8 (12.2)
BMI, Kg/m^2^: Mean (SD)	27.3 (4.41)	30.3 (2.8)	27.9 (5.7)	26.6 (3.6)
Menopause	13 (54.2%)	1 (25%)	3 (50%)	9 (64.2%)
Diabetes	2 (8%)	0%	0%	2 (14.3%)
Family history of cancer	11 (45.8%)	2 (50%)	4 (66.7%)	5 (35.7%)
Type of cancer				
Triple negative breast cancer	14 (58.3%)	2 (50%)	3 (50%)	9 (64.3%)
Early onset breast cancer	2 (8%)	0%	1 (16%)	1 (7%)
High grade serous carcinoma	6 (25%)	2 (50%)	2 (33.3%)	2 (14.3%)
Endometroid carcinoma	2 (8%)	0%	0%	2 (14.3%)

## Discussion

Genetic testing for germline *BRCA *mutation in breast cancer patients provides important information not only for prognosis and treatment but also provides candidates for genetic counseling from the patient’s family to know their potential risk for the breast cancer and early diagnosis by doing genetic testing of that particular pathogenic variant. In our study, we have screened 24 Pakistani cases for germline mutations causative for breast and ovarian cancer subtypes by doing targeted sequencing of *BRCA1/BRCA2* genes, with moderate family history provided. 

Quality scores of this NGS panel depicted successful execution of germline genetic testing of the study participants. And the variants having quality score of 100 were proceeded for further analysis and interpretation. All *BRCA1/2* variants detected were previously reported in the external databases; ClinVar, dbSNP, The* BRCA *exchange and GDC portal, and no novel mutation found in any case under study. 

Germline *BRCA* mutations are highly penetrant in breast or ovarian cancer, which run as autosomal dominant form. Most cases of *BRCA* mutated breast cancer are sporadic than familial.* BRCA1* mutations are more likely to cause TNBC than *BRCA2* mutations (Tun et al., 2014). A prospective study analyzed 2733 women in the United Kingdom from 2000 to 2008, illustrated the associated of *BRCA* mutations in sporadic versus hereditary breast cancer. Overall, 12.4% women had germline* BRCA *mutation that includes 5.4% *BRCA2* and 7.4% *BRCA1* mutations (Copson et al., 2018). In a comprehensive study of genetic testing of 192 TNBC cases in Pakistan, Sanger sequencing method was used for screening of 26 hotspot mutations in *BRCA1* and *BRCA2*. 125 cases with pathogenic mutations were identified, in which 84% variants are of* BRCA1* 16% of* BRCA2* detected (Rashid et al., 2016). Referring to Table 1, our study reported all pathogenic variants in TNBC cases in *BRCA1* gene with no clinically significant variant in *BRCA2*, complementing the previous studies mentioned above.

Approximately, 10–15% of epithelial ovarian cancer (EOC) patients carry germline mutation in* BRCA1* or *BRCA2*. (Zhang et al., 2011; Alsop et al., 2012), and it was particularly high in high grade serous subtype, which was reported at about 20–30% (Network, 2011). Incidence of *BRCA* mutation in high grade serous subtype were reported as high as 30–40% (Wu et al., 2017). In our study, we found 33.3% (n = 6) *BRCA1* pathogenic variants in high grade serous carcinoma which is in the range of foreign studies. According to a systematic review, endometroid subtype had a lower probability of having germline *BRCA* variant, reported about 7.7% (Arts-de Jong et al., 2016). And in our study, clinically significant *BRCA* variant was not detected in patients with endometrioid carcinoma.

Studies showed that in cases without family history of breast and/or ovarian cancers, the incidence of *BRCA* mutation was reported about 10%. In contrast, 60–70% of cases with family history of cancers had *BRCA* variant. (Wu et al., 2017; Manchana et al., 2019). Due to limitations of our sample size, we detected 18.2% *BRCA* pathogenic mutations in cases with family history of cancer. 

In our study, reportable *BRCA* variants have been detected in TNBC cases aged less than 50, interpreted according to the ACMG guidelines, complementing other ethnic cohort studies. In a German consortium study, they concluded that every triple negative breast cancer patient aged less 50 should be screened for germline *BRCA* mutations regardless of family history (Engel et al., 2018). About 50% of Israeli EOC patients younger than 50 years carried BRCA variant (Helpman et al., 2017). EOC cases, aged more than 50, carried *BRCA* mutation in our study. 

The limitations in this study were; the sample size was not calculated as per the standard guideline because this study was dependent on limited resources and there is no data published from Pakistan on *BRCA* testing by NGS, giving valuable information to the families under study. 

The incidence of germline mutation in breast cancer and ovarian carcinoma subtypes in this study was 12.5% and 25% respectively. However, all *BRCA1* mutation was found in Triple negative breast cancer and high-grade serous subtype, none in *BRCA2*. Although, various societies such as American College of Obstetricians and Gynecologists (ACOG), Society of Gynecologic Oncologists (SGO), and National Comprehensive Cancer Network (NCCN) have suggested universal genetic counseling and testing for all TNBC (aged ≤ 60) and EOC patients (Alberts and Hess, 2019). High cost, limited number of geneticists, and unavailability of genetic testing may be the barriers in limited resource countries. To improve knowledge and increase patient awareness, it is one major challenge besides limited number of genetic testing centers in Pakistan. 

In conclusion, considering the high incidence of breast and ovarian carcinoma in Pakistani subset of population, it is imperative to find the frequency of hereditary BRCA mutation in these carcinomas .It will not only help in initiating targeted therapy but will also provide candidates for familial screening .Our study demonstrated a high percentage of hereditary mutation in cancer subset further demonstrating the need for extensive testing. Also the positive correlation between BRCA mutation and early onset breast and ovarian carcinoma was evident from the results of the current study.

## Author Contribution Statement

H.T jointly conceived the study with A.G and H.U.D, designed and executed this project with T.K, H.N.T and M.A. A.G and H.T interpreted NGS data and wrote the manuscript. R.A and M.A helped in case selection and in wet lab. H.N.T and R.A assisted in statical analysis. T.K and H.U.D supervised the project. 
